# Honey as a Cytological Fixative: A Comparative Study With 95% Alcohol

**DOI:** 10.7759/cureus.28149

**Published:** 2022-08-18

**Authors:** Avinash Priyadarshi, Rupinder Kaur, Roma Issacs

**Affiliations:** 1 Pathology, Mata Gujri Memorial (MGM) Medical College & Lions Seva Kendra (LSK) Hospital, Kishaganj, IND; 2 Pathology, Christian Medical College & Hospital, Ludhiana, IND; 3 Pathology, Maharishi Markandeshwar Institute of Medical Science and Research, Ambala, IND; 4 Pathology, Christian Medical College & Brown Memorial Hospital, Ludhiana, IND

**Keywords:** papanicolaou stain, honey, ethanol, cytomorphology, cytological fixatives

## Abstract

Background

Ninety-five percent (95%) ethyl alcohol (ethanol) has been used as a standard cytological fixative but it is expensive, difficult to procure, and has addictive properties. Alternate substitutes like methanol, which give similar results to ethanol, have toxic potential. Honey, a known preservative, is an eco-friendly fixative and is of great value when ethanol is unavailable and economizing on cost is necessary. The present study was done to assess and compare the fixation property and cytomorphological features of smears fixed in 20% honey in comparison to 95% ethyl alcohol and to determine whether the former can be used as an alternative cytological fixative in routine practice.

Material and methods

The present prospective study was done in the cytology section of the Department of Pathology for one and a half years on 300 cytological samples comprising 100 samples each of various body fluids (peritoneal, pleural, bronchoalveolar lavage, and urine), cervical smears, and fine-needle aspiration samples. The smears from all the 300 cytological samples were fixed separately in 95% ethanol and 20% unprocessed natural honey for a minimum of 15 minutes and were then stained with Papanicolaou (Pap) stain. The cytomorphological parameters of both the smears were compared based on set criteria. Relevant statistical analysis was done using the student t-test, chi-square test, and test of agreement (kappa statistics).

Results

A comparable and good-quality staining pattern, preservation of morphology, and crisp nuclear and cytoplasmic staining were observed between the two fixatives for all three types of samples with a strong agreement between them (kappa value varying between 0.896 and 0.942) and a p-value of <0.05.

Conclusion

Natural honey is a readily available and non-toxic alternative to ethanol as a cytological fixative and can be used in routine practices, especially in resource-constrained settings.

## Introduction

Fixatives play a very important role in cytopathology apart from the quality of material collected and its interpretation for an accurate and reliable diagnosis. A discrepancy in any of these steps has an adverse effect on the final diagnosis. Ninety-five percent (95%) ethyl alcohol (ethanol) has been used as a standard cytological fixative but is difficult to procure and has addictive properties [1]. Alternative fixatives like methanol are known to have a toxic potential [2-3]. Hence, there is a need for readily available and eco-friendly fixatives, which have equivalent or similar properties to conventional fixatives. Unprocessed honey, a known preservative, has been shown to have comparable results as those obtained from conventional formalin-fixed control tissues on Hematoxylin and Eosin (H&E) stain and hence can be of great value when ethanol is difficult to procure [4-5].^ ^A thorough and detailed literature search showed a few studies with limited cases and study material comparing unprocessed honey as an alternative fixative in cytology [6-9].

The present study was done to compare the cytomorphological parameters between smears fixed in 95% ethanol and 20% unprocessed honey on Papanicolaou (Pap) stain.

## Materials and methods

This study was done in the Cytology section of the Department of Pathology in a tertiary care center in North India. This was a prospective study done over a period of one and a half years on smears made from 300 cytological samples comprising 100 samples each of various body fluids (peritoneal, pleural, bronchoalveolar lavage, and urine), cervical smears, and fine-needle aspiration samples. The study was approved by the Faculty of Medical Sciences of the respective university (Letter No BFUHS/2K-16p-TH/7999, dated 19/7/16).

Unprocessed honey (commercial) used in our study was procured from the market in the smallest of packing (50 gm). Once opened, the same was kept in the refrigerator (2-8^0^C) for a maximum of one week.

Samples from the fluids were centrifuged at 1500 rpm for 30 minutes and smears were made from the sediment. Fine-needle aspiration was done on various lesions on the patients referred to the cytology laboratory with the help of a 21-gauge needle with a 20 ml syringe fitted on to Franzen syringe holder. Smears were made from the material collected both from the syringe and the hub of the needle.

Apart from the routine smears, additional two smears were also made both from the body fluids as well as the fine-needle aspiration cytology (FNAC) material. The respective additional smears were labeled A and B and were fixed in 95% ethanol and 20% unprocessed honey, respectively, for a minimum of 15 minutes. Fictitious numbers were given on the smears to be evaluated so as to avoid observer bias.

**Figure 1:**
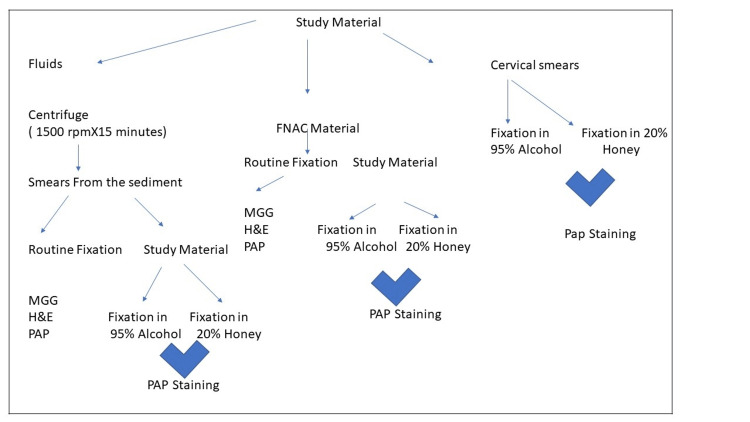
Flow chart depicting details of sample processing FNAC: fine-needle aspiration cytology

The cervical smears prepared by the gynecologists in the Gynaecological outpatient department (Gynae OPD) were immediately wet-fixed in 95% ethanol as a part of routine processing and were labeled A. An additional smear was also taken (labeled B) and was immediately wet-fixed in 20% unprocessed honey (Figure 1).

All the smears, including the conventional as well as the additional smears (A&B) prepared, were stained with Papanicolaou (Pap) stain after the designated fixation period. The stained smears to be compared from both the fixatives were given a fictitious number by covering the original number with a sticker so as to avoid observer bias. The stained smears were seen independently by two pathologists, so as to maintain uniformity in smear assessment.

The paired smear was evaluated independently based on five parameters through a scoring system based on the modified criteria given by Singh et al. as listed in Table 1 [6].

**Table 1:** Modified evaluation criteria given by Singh et al. based on various features Source: [6]

Features	Scores	Criteria	
Clarity of staining	Score 1	Crisp and transparent staining	Present and adequate
	Score 0	Obliteration of nucleus and cytoplasmic staining	Absent and inadequate
Uniformity of staining	Score 1	Homogenous staining throughout the cells	Present and adequate
	Score 0	Different shades of color in individual cells	Present and inadequate
Overall morphology	Score 1	Absence of folds, overlapping, or nuclear swelling	Preserved and adequate
	Score 0	Disintegrated cells with overlapping and folding	Unpreserved and inadequate
Nuclear details	Score 1	Round nuclei with smooth and clear nuclear membrane	Acceptable and adequate
	Score 0	Nuclear granularity and disintegration	Unacceptable and inadequate
Cytoplasmic details	Score 1	Intact cytoplasmic membrane with transparent cytoplasm	Acceptable and adequate
	Score 0:	Disintegrated cytoplasmic membrane with out-of-focus granular cytoplasm	Unacceptable and inadequate
Total score/ grade	Score 5:	Excellent	
	Score 3-4:	Good	
	Score ≤ 2:	Poor	

The total score was obtained by adding each parameter and grading all the slides. Decoding of the slides was done thereafter with the diagnosis made based on the morphological parameters. The same was then compared with the final diagnosis based on the conventional smears.

The final results were subjected to statistical analysis for the student’s t-test, chi-square test, and test of agreement (kappa statistics).

## Results

The study included 300 cytological samples comprising 100 samples each of various body fluids, (peritoneal, pleural, bronchoalveolar lavage, and urine), cervical smears, and fine-needle aspiration samples. The details of the distribution of the total number of samples are given in Table 2.

**Table 2:** Detailed list of the total number of samples processed BAL: bronchoalveolar lavage

Sample	Site	Number	Total
Fluid	Urine	34	100
	Pleural Fluid	28	
	Ascitic Fluid	16	
	BAL	8	
	Sputum	7	
	Peritoneal Fluid	3	
	Pericardial Fluid	2	
	Synovial Fluid	2	
Cervical Pap	Cervix	100	100
FNAC	Lymph Node	48	100
	Breast Lump	28	
	Thyroid	13	
	Testis	3	
	Epididymal Cyst	1	
	Parotid Gland	1	
	Spleen	1	
	Liver	1	
	Toe Swelling	1	
	Thigh Lump	1	
	Leg Swelling	1	
	Scapular Swelling	1	

The cytological parameters of both the smears (A&B) were evaluated based on the parameters given by Singh et al. [6] and revised criteria for the scoring system were given. Diagnosis made on both the smears was then compared with the smears prepared through the conventional method. To determine the degree of agreement between the two fixatives, a measure of agreement - kappa - was utilized and the p-value was calculated. The details of the results based on the criteria are given in Table 3.

**Table 3:** Comparison and correlation between various parameters among study sample types FNAC: fine-needle aspiration cytology

Sample type	Parameters		Percentages		Kappa value	p-value
			Conventional	Honey (20%)		
Fluid (Figures 2A-2D and Figures 3A-3B)	Clarity of the staining	Adequate	84	81	0.896	0.577
		Inadequate	16	19		
	Uniformity of the staining	Adequate	88	87	0.945	0.831
		Inadequate	12	13		
	Nuclear details	Adequate	92	94	0.847	0.579
		Inadequate	08	06		
	Cytoplasmic details	Adequate	92	93	0.784	0.788
		Inadequate	08	07		
	Overall morphology	Adequate	93	92	0.928	0.788
		Inadequate	07	06		
Cervical smear (Figures 3C-3D and Figures 4A-4B)	Clarity of the staining	Adequate	95	94	0.904	0.756
		Inadequate	05	06		
	Uniformity of the staining	Adequate	95	93	0.823	0.552
		Inadequate	05	07		
	Nuclear details	Adequate	89	90	0.878	0.602
		Inadequate	11	10		
	Cytoplasmic details	Adequate	92	90	0.947	0.818
		Inadequate	08	10		
	Overall morphology	Adequate	93	91	0.864	0.621
		Inadequate	07	09		
FNAC (Figures 4C-4D, Figures 5A-5D, and Figures 6A-6D)	Clarity of the staining	Adequate	94	93	0.918	0.774
		Inadequate	06	07		
	Uniformity of the staining	Adequate	91	90	0.942	0.809
		Inadequate	09	10		
	Nuclear details	Adequate	96	91	0.918	0.774
		Inadequate	04	09		
	Cytoplasmic details	Adequate	93	94	0.884	0.733
		Inadequate	07	06		
	Overall morphology	Adequate	94	93	0.918	0.774
		Inadequate	06	07		

**Figure 2:**
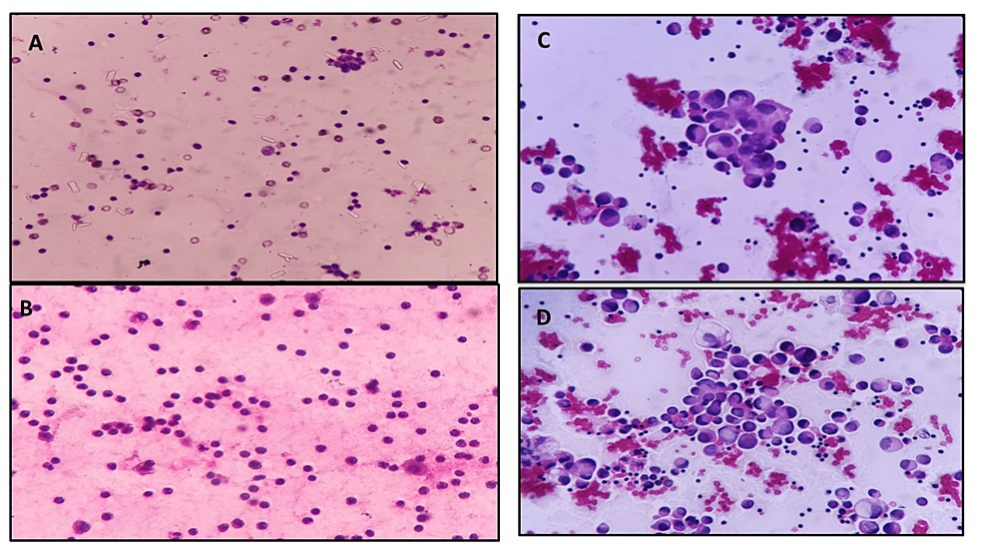
Microphotograph of lymphocytic pleural effusion smears fixed in 95% alcohol (A) and 20% honey; (B); reactive mesothelial cells in pleural fluid smears fixed in 95% alcohol (C) and 20% honey (D)

**Figure 3:**
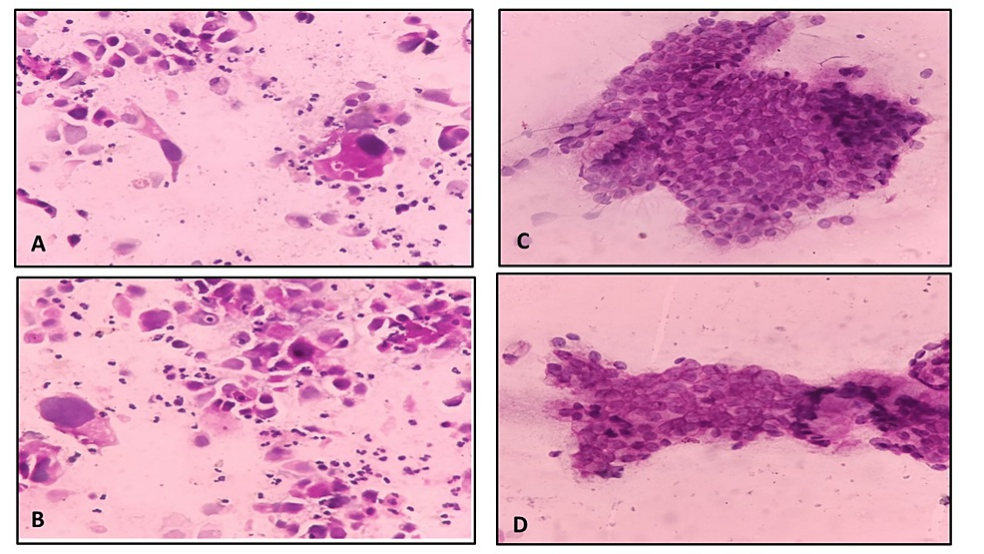
Microphotograph of Pap-stained smears of urine showing malignant cells in smears fixed with 95% alcohol (A) and 20% honey (B); endocervical cells in smears fixed in 95% alcohol (C) and 20% honey (D) Pap: Papanicolaou

**Figure 4:**
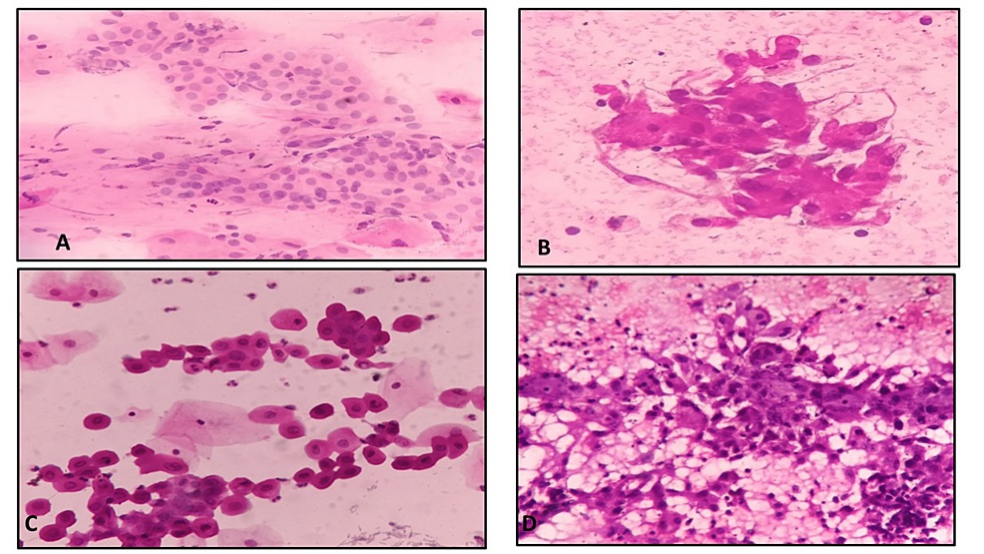
Microphotograph showing atrophic changes in the cervix in smears fixed with 95% alcohol (A) and 20% honey (B); apocrine cells in breast FNAC smears fixed in 95% alcohol (C) and 20% honey (D) FNAC: fine-needle aspiration cytology

**Figure 5:**
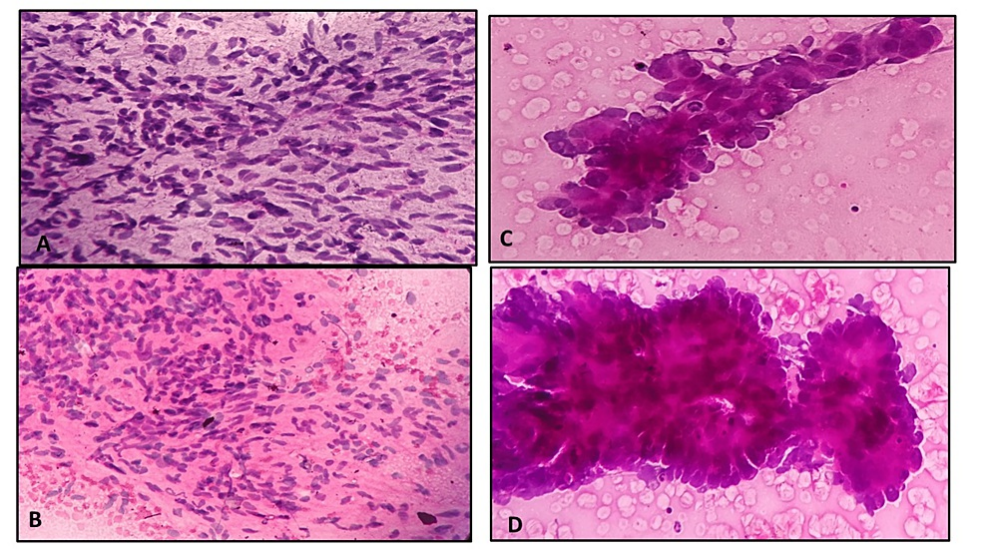
Microphotograph of FNAC breast showing a phyllodes tumor in smears fixed with 95% alcohol (A) and 20% honey (B); metastatic adenocarcinomatous deposits, liver FNAC smears fixed in 95% alcohol (C) and 20% honey (D) FNAC: fine-needle aspiration cytology

**Figure 6:**
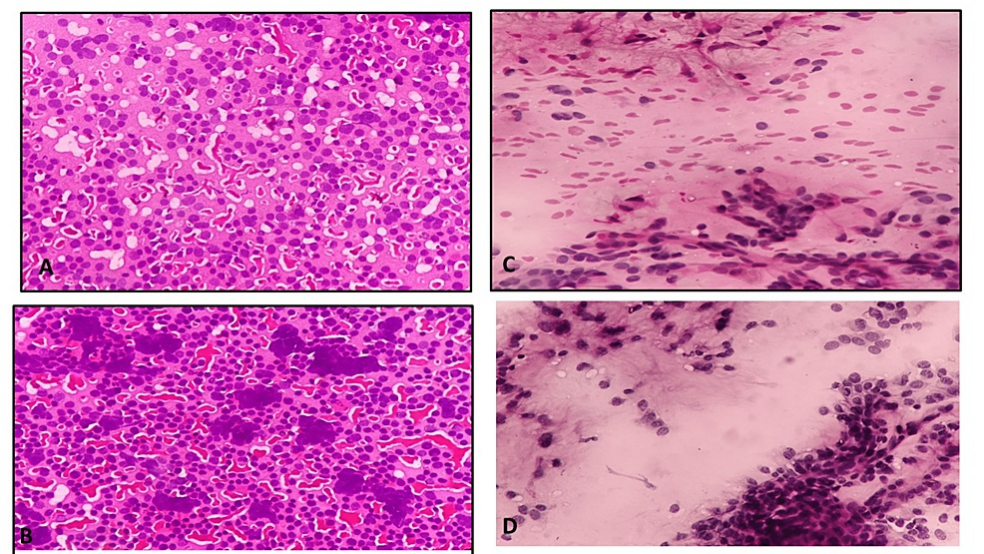
Microphotograph of FNAC lymph node showing NHL in smears fixed with 95% alcohol (A) and 20% honey (B); FNAC of parotid gland showing pleomorphic adenoma in smears fixed with 95% alcohol (C) and 20% honey (D) FNAC: fine-needle aspiration cytology

Microscopic images showed comparable qualities based on modified criteria given by Singh et al. [6] for body fluids (Figures 2A-2D and Figures 3A-3B), cervical smears (Figures 3C-3D) and Figures 4A-4B) and FNAC material (Figures 4C-4D), Figures 5A-5D, and Figures 6A-6D).

A strong correlation was observed between various parameters among both the fixatives in all the sample types. There was no statistical difference between the fixative properties of alcohol and honey (Table 4).

**Table 4:** Comparison of the overall score and grade among various sample types FNAC: fine-needle aspiration cytology

Sample type	Kappa value		p-value	
	Overall score	Overall grade	Overall score	Overall grade
Fluid	0.791	0.800	0.911	0.777
Cervical smear	0.834	0.851	0.685	0.465
FNAC smear	0.885	0.880	0.987	0.877

## Discussion

Over the years, alcohol in various forms (ethanol, methanol) has been used as fixatives for the preservation of cellular details, thus aiding in cytological assessment and diagnosis. However, there is always an issue with their availability and procurement, as they are subjected to pilferage, have addictive and carcinogenic properties, are inflammable, irritate the skin and eye, evaporate easily, and most importantly, they require a license [1]. A need for an alternative natural substitute that is readily available, has fewer biohazardous properties, and has fixative and staining qualities equivalent to alcohol fixatives led to the use of unprocessed honey and implicating its role as a fixative. Apart from being a good fixative, honey also has antimicrobial activity and prevents autolysis and putrefaction [5,6,10-11].

The present study was undertaken to assess the potency of unprocessed honey (20%) as a fixative for cytological smears from various sites (body fluids, cervical smears, and FNAC material) and compared it with the smears fixed in 95% ethanol. A detailed literature search revealed few studies comparing honey as an alternative cytological fixative [3,5-9,12-16]. The details of the comparison between various studies are given in Table 5.

**Table 5:** Showing comparative analysis of various studies FNAC: fine-needle aspiration cytology; NBF: neutral buffered formalin

Studies	Alternative fixative used	Total sample size	Types of smears	Statistical analysis: (Kappa value/Kruskal- Wallis test/p-value)
Kumarasinghe MP et al (1997) [3]	Methanol	108	FNA of thyroid	p > 0.05 (NS)
Ozkan et al (2012) [4]	10% honey NBF and alcoholic formalin	7	Tissue samples each from the endometrium, breast, placenta, uterus, omentum, suprarenal, stomach, and lung	p > 0.05 (NS) 10% honey and alcoholic formalin), p <0.05 (S), 10% honey and NBF
Sabrinath et al ( 2014) [5]	Formalin + honey	13 (formalin-fixed tissue) & 17 (honey-fixed tissue	Maxillofacial tissue	p-value < 0.05 (S)
Singh A et al (2015) [6]	20% honey	30	Buccal smears	Kruskal-Wallis test ( X^2^ ): 1.10, p-value: 0.47 (NS)
Lalwani et al (2015) [12]	20% processed honey+ 20% unprocessed honey + formalin	36	Human tissue (oral epithelium, lymphoid, salivary gland, fat, muscle, and skin	p-value = 0.04 (NS)
Ishaq R et al (2016) [7]	20% honey	30	FNAC sample	p-value > 0.05 (NS)
Sona M et al (2017) [8]	20% honey	194	Buccal smears of healthy individuals	Kappa value: 0.879, p-value: 0.842 (NS) (overall staining quality)
Pandiar D (2017) [9]	20% honey and 30% aqueous jaggery solution	25	Oral smears of healthy individuals	p-value > 0.05 (NS)
Kuriachan et al (2017)^13^	Honey, jaggery, and sugar compared with formalin	40	Human gingival tissue	p-value: <0.05 (S); honey and jaggery gave superior results
Khan et al (2018) [14]	20% honey	200	Buccal smears	p-value: >0.05 (NS)
Nerune et al (2019) [15]	20% processed honey	50	Buccal mucosa	p-value: >0.05 (NS)
Sah et al (2022) [16]	20% processed honey and 20% jaggery	60 (healthy subjects)	Buccal mucosa	Kruskal-Wallis test ( X^2^ ): 4.93 p-value = 0.41 (NS)
Present study	20% unprocessed honey	300 (100 each)	Fluid (F) + cervical smears (CS) + FNAC (FN) smears	Kappa value: overall grade F:0.800, CS:0.851, FN:0.880, p-value: Overall grade (NS). F: 0.777, CS: 0.465, FN: 0.877

The studies done by the above-mentioned authors were limited to one particular site or procedure with smaller sample size. In contrast to this, the present study had material from various sites with a sufficient sample size (300 paired samples) for comparison and evaluation. The paired smears from all the samples collected were evaluated for five parameters, viz. clarity of staining, uniformity of staining, overall morphology, and nuclear and cytoplasmic details. Slides were scored for the parameters based on modified criteria given by Singh et al. [6].

Singh et al. in their study showed that 3% of honey-fixed slides and 10% of ethanol-fixed slides had unacceptable nuclear staining, which was mainly attributed to eosinophilic staining of the nuclei in ethanol-fixed and light staining of the same in honey-fixed smears [6]. In their study, they also found that the size and shape of the cells were better in honey-fixed smears, whereas the clarity and uniformity of the staining were much better in ethanol-fixed smears but no statistical difference was observed when overall scores were taken into account. However, a study done by Ishaq et al. showed a statistically significant difference (p=0.006) only in the clarity of staining between the two fixatives whereby smears fixed in ethanol showed better clarity, which was attributed to the viscous nature of honey [7]. The cytoplasmic staining was not transparent and there was granularity due to the constituents of honey. Similar findings were also observed in our study, especially in cervical smears; however, the percentage of such material was negligible, was not uniformly seen in the entire smear, and finally did not hinder the final diagnosis of the smears.

In contrast to this, a study done by Pandiar et al. showed better clarity of staining with honey-fixed smears [9]. They found honey to be slightly better than ethanol and jaggery when nuclear and cytoplasmic staining characteristics were compared. However, there was no overall statistical difference between the three fixatives. The study done by Sona et al. on buccal smears also showed no statistically significant differences (p>0.05) between the two fixatives based on the above-mentioned cytological parameters [8].

Singh et al. in their final analysis of all scores revealed that 90% of ethanol-fixed and 80% of honey-fixed smears were adequate for analysis; however, no statistical difference was seen between the two fixatives [6]. In addition to this, they also found that the honey-fixed smears had a clearer background as compared to the ethanol-fixed smears; however, no such difference was seen in the present study. Sona et al. showed a kappa value for the overall score for staining quality to be 0.879, which was in strong agreement between the two fixatives. This was in concordance with our study.

Limitations

There were a few limitations with regard to using honey as a fixative. It had a decreased shelf life, as it attracted insects and caused mold formation when left over a period of time, which was solved by refrigeration and frequent change of the alternative fixative within two to three days. It also caused loss of material during wet fixation; however, this was avoided by drying the smear for a few seconds.

## Conclusions

The present study was done to compare both conventional and honey-fixed smears. The cytological parameters for the adequacy of the diagnosis were analyzed. There was no statistically significant difference between the two fixatives. The overall score and grading were also comparable. Based on all the observations from the present study, it was concluded that unprocessed honey had all the properties that an ideal fixative should have: it is easily available, non-toxic, eco-friendly, and can be used as an alternative fixative to ethanol for routine purposes.
